# A survey on the orthopedic and functional assessment in a Portuguese population of police working dogs

**DOI:** 10.1186/s12917-022-03221-8

**Published:** 2022-03-25

**Authors:** João Carlos Agostinho Alves, Patrícia Isabel Figueiredo Jorge, Ana Margarida Moniz Pereira dos Santos

**Affiliations:** 1 Divisão de Medicina Veterinária, Guarda Nacional Republicana (GNR), Rua Presidente Arriaga, 9 1200-771 Lisbon, Portugal; 2 MED–Mediterranean Institute for Agriculture, Environment and Development, Instituto de Investigação E Formação Avançada, Universidade de Évora, Pólo da Mitra, Ap. 94, 7006-554 Évora, Portugal

**Keywords:** Police working dog, Musculoskeletal disease, Canine Orthopedic Index, Functional Assessment, Hudson Visual Analogue Scale

## Abstract

**Background:**

Working dogs are at an increased risk of developing an orthopedic disease compared to companion dogs. This study aimed to evaluate functional and orthopedic index fitness in a Portuguese population of police working dogs.

In an observational, prospective study, information on 165 dogs was collected. The age, sex, breed, specific work, and history of previous diagnosis of orthopedic disease were recorded for each patient. A copy of the Canine Orthopedic Index (COI), Hudson Visual Analogue Scale (HVAS), and Functional Assessment (FA) was collected for all dogs. COI, HVAS, and FA scores between breeds, work, age, sex, and history of a previous diagnosis of orthopedic disease were compared. Multiple regression was run to predict COI, HVAS, and FA scores from breeds, work, age, sex, and history of orthopedic disease. Correlations between items were determined with Pearson's correlation. A *p* < 0.05 was set.

**Results:**

The sample was composed of 92 males and 73 females, with a mean age of 5.2 ± 3.2 years. Four main dog breeds were represented, 60 Belgian Malinois Shepherd Dogs, 52 German Shepherd Dogs, 29 Labrador Retrievers, and 14 Dutch Shepherd Dog. A prevalence of diarrhea of 10.6% was determined, with 4% of dogs having liquid diarrhea. German Shepherd Dogs had significantly higher FA scores (*p* = 0.03). Dogs with a history of previous veterinary assistance due to orthopedic issues had significantly lower HVAS scores and higher scores with all remaining questionnaires (*p* < 0.01 for all). No differences were found between sexes or specific work. Age and a history of orthopedic disease contributed to the prediction of all scores. FA scores had a good correlation with COI and HVAS.

**Conclusion:**

This population of police working dogs has a good to excellent level of physical fitness. There was a relationship between increasing age, history of orthopedic disease, and worse scores with all questionnaires. All considered questionnaires could differentiate between animals with a previous history of orthopedic disease and sound dogs.

## Background

In police forces, working dogs are commonly employed in several tasks, including search and rescue, product detection (drugs, explosives, and others), use of force, and others [1]. As sporting dogs, working dogs are at an increased risk of developing an orthopedic disease than companion dogs [2–4] due to some breed predispositions and the increased stress related to the activities they are engaged in [2]. Lameness is a common condition for early retirement in police working dogs, amounting to up to 69% of cases in some reports [3, 4]. Some of the most common causes of lameness are osteoarthritis [5] and degenerative lumbosacral disease [2, 6]. In an attempt to address those problems, canine working dogs training programs have tried to introduce measures that prepare dogs from an early age to overcome obstacles in an adequate way [7] and promote physical conditioning and preparation [8–10].

In addition to preventing orthopedic disease, there is a need to detect early cases to improve veterinary intervention ability and maximize outcomes [11]. Besides an orthopedic examination that typically involves a stage of gait evaluation and a hands-on examination [12], there is also a need to have valid and reliable quantitative outcome measures to improve diagnosis quality and monitor interventions [13]. Clinical metrology instruments can aid in this process and are constituted by a questionnaire that comprises several questions or items, scored according to a proxy's (in this case, the handler) experiences or observations. The individual scores are then used to calculate an overall instrument score [14–16]. There is not a wide availability of orthopedic instruments to perform this evaluation [17]. The Canine Orthopaedic Index (COI) is a validated tool developed to assess four domains in dogs: stiffness, gait, function, and quality of life. It is a 4 factor, 16 item questionnaire designed to measure owner assessment of those four domains. It was shown to have excellent reliability and validity. It was also shown to differentiate sound from sick animals [18, 19] and evaluate response to treatment [13]. A functional assessment scale (FA), while still not validated, has also been developed to assess the presence of degenerative lumbosacral disease and response to treatment [6, 17]. The Hudson Visual Analogue Scale (HVAS) has been compared with force plate analysis. It was shown to be repeatable and valid to assess the degree of mild to moderate lameness [20].

This study aimed to evaluate functional and orthopedic index fitness in a Portuguese population of police working dogs and evaluate the relation between the level of fitness with animals' age, breed, sex, specific work, and a previous diagnosis of orthopedic disease. We hypothesized that these factors would influence the observed scores.

## Results

Information on 165 animals was collected, with a mean age of 5.2 ± 3.2 years, representing both sexes (92 males and 73 females). Four main dog breeds were represented, 60 Belgian Malinois Shepherd Dogs, 52 German Shepherd Dogs, 29 Labrador Retrievers, 14 Dutch Shepherd Dog, and 10 animals from other breeds. Regarding specific work, 80 (48.5%) animals were use of force dogs, 68 (41.2%) were product detection dogs (drugs or explosives), and 16 (10.3%) were search and rescue dogs. Fifty-three (32.1%) animals had received previous veterinary assistance due to an orthopedic condition. Overall HVAS, COI, and FA scores, by breed, sex, specific work, and history of orthopedic or neurological disease are presented in Table 1 . Significant differences were found between breeds considering FA scores, with German Shepherd Dogs showing higher FA scores (*p* = 0.03). Dogs with a history of previous veterinary assistance due to orthopedic issues had significantly higher COI and FA scores and significantly lower HVAS scores (*p* < 0.01 for all). No differences were found between sexes or specific work.

**Table 1:** Mean values and standard deviation of overall Hudson Visual Analogue Scale (HVAS), Canine Orthopedic Index (COI), and Functional Assessment (FA) and by breed, sex, specific work, and history of orthopedic or neurological disease. QOL – quality of life

	HVAS (0–10)		Canine Orthopedic Index										FA (0–40)	
			Stiffness (0–16)		Gait (0–20)		Function (0–16)		QOL (0–12)		COI (0–64)			
	mean	SD	mean	SD	mean	SD	mean	SD	mean	SD	mean	SD	mean	SD
Overall	5.3	6.9	1.8	3.0	2.6	4.3	1.5	3.1	2.5	2.9	8.4	12.4	5.3	6.9
Belgian Malinois Shepherd Dog	7.9	1.3	1.5	2.8	1.2	2.9	2.2	4.2	2.2	2.8	7.0	12.0	3.6	5.7
German Shepherd Dog	7.5	1.4	2.6	3.3	2.2	3.6	4.1	4.9	3.3	3.1	12.2	14	7.6	8.4
Labrador Retriever	7.9	1.4	1.6	3.1	1.1	2.9	1.8	3.7	2.2	2.7	6.7	11.7	5.8	5.8
Dutch Shepherd Dog	7.8	1.2	0.9	1.8	0.9	1.8	1.1	2.4	2.1	1.7	4.9	6.6	3.7	4.3
Male	7.8	1.3	1.9	2.9	1.6	3.2	2.7	4.3	2.3	2.8	8.5	12.6	5.7	7.2
Female	7.8	1.3	1.6	3.1	1.3	2.9	2.4	4.2	2.8	2.9	8.2	12.1	4.7	6.3
Use of force	7.8	1.3	1.9	3.1	1.5	3.2	2.6	4.3	2.5	2.9	8.5	12.7	4.8	6.9
Production detection	7.9	1.3	1.7	3.0	1.5	3.3	2.5	4.3	2.4	2.8	8.1	12.5	5.7	6.9
Search and rescue	7.5	1.4	1.6	2.3	1.3	1.9	2.9	3.9	3.2	2.7	8.9	9.8	5.7	6.1
History of disease	6.9	1.6	4.1	3.6	3.7	4.3	6.1	5.4	4.7	3.0	18.6	15.1	10.2	8.7
Without history of disease	8.2	0.9	0.7	1.8	0.4	1.5	0.9	2.2	1.5	2.1	3.5	6.6	2.9	4.1

Breed significantly contributed to the prediction of gait scores F(1, 157) = 4.231, *p* = 0.04, R2 = 0.025. Age also contributed to the prediction of all scores F(7, 158) = 113.669, *p* < 0.01, R2 = 0.000, as did a history of previous orthopedic disease F(6, 158) = 13.380, *p* < 0.01, R2 = 0.337. A correlation was observed between a history of previous veterinary assistance due to orthopedic issues and all scores (*p* < 0.01 for all), being negative for HVAS (-0.429) and positive for the remaining scores (ragging from 0.493 and 0.570). FA scores showed a high correlation with COI and HVAS scores (*p* < 0.01 for all, with -0.701 for HVAS and ragging between 0.839 and 0.864 for the COI domains).

## Discussion

Working dogs have an important role in police forces throughout the world, and lameness is a common condition for the early retirement of these animals. This study assessed functional and orthopedic fitness in a Portuguese population of police working dogs. Our results showed that these dogs have a good to excellent overall quality of life, being able to carry out their specific work.

The COI has been used before to assess companion animals and working dogs [18, 21]. Our results showed that the dogs from this population had low scores across breeds, sex, and specific work. These results are in line with previous reports on police working dogs [17]. This is not completely surprising, as these animals are routinely evaluated and go through certification processes. Their ability to go through these processes would be considerably impaired in the presence of disease. The COI was not constructed as a tool to survey dogs for orthopedic disease, although it has been used to validate other screening and evaluation modalities [22]. Still, our results showed that dogs with a previous history of veterinary assistance due to orthopedic disease have significantly higher scores in all domains. Although these animals still had a fitness level suitable to maintain an active duty, probably associated with adequate veterinary assistance, the COI could differentiate these animals from sound ones. HVAS scores were also high, showing a good level of mobility. In addition, as dogs with a previous history of orthopedic disease accounted for only 32.1% of all dogs surveyed, in line with previous reports [17], it is reasonable to assume that a large majority of animals in this population have an excellent fitness level.

With FA, scores between 5 and 7 are classified as showing evidence of borderline impairment, and scores > 8 were classified as functionally impaired [17]. Some of the breeds considered showed a level of borderline impairment, notably German Shepherd Dogs, at a significantly higher level than the remaining breeds considered. Despite being a breed widely presented in police and military forces worldwide, weakness in the pelvic limbs due to lumbosacral disease or osteoarthritis have been reported in working German Shepherd Dogs [2, 23, 24]. FA scores seemed to be relatively higher than those observed with COI, and this finding may be related to the nature of the questions presented. Some FA questions address the frequency of difficulties in overcoming specific obstacles or exercises. As these exercises may require some training, and although instructions were provided in that sense, some handlers may have considered a difficulty in overcoming an obstacle due to a lack of proper training or training at an early stage, with a physical difficulty to do so. Subsequent studies should address this question. Part of the assessment of construct validity of a new instrument can be performed by comparing the results of an instrument to a validated one [14, 25, 26]. With that in mind, it is relevant to see that FA scores correlate with COI and HVAS, two previously validated instruments. Although this supports the use of FA, it is not sufficient to prove it is a validated questionnaire. One could question if considering a single instrument would be sufficient to assess patients. Orthopedic and neurological diseases are characterized by variable degrees of clinical and functional impairments that affect the various aspects of a patient's life differently [27]. With that in mind, applying different instruments may help capture these different aspects.

As all of these instruments are completed by a proxy, the proxy's ability to properly perform the assessment can influence the results of the instrument [28, 29]. Although potential limitations may occur, in this study, the instruments were completed by dog handlers. It is reasonable to consider that they have a good sensibility to evaluate the dog's demeanor and overall activity.

Age and history of orthopedic disease were the factors that significantly contributed to the prediction of all scores considered. This finding is in line with previous reports, where increasing age coincides with worse questionnaire scores and responses to treatment [5, 17, 30]. The two may be associated as, on the one hand, as the time of active work increases, the possibility for injury also increases. On the other hand, increasing age is also related to diagnosing some orthopedic diseases, such as osteoarthritis [31, 32].

This study presents some limitations, namely that the severity of previous orthopedic disease was not determined. It is also possible that some dogs may have undiagnosed conditions. Still, based on the nature of the veterinary assistance available to these dogs and the overall low scores observed, it is reasonable to assume that cases of severe disease are unlikely. An additional limitation is a lack of an objective measure of lameness, such as force plait gait analysis. Although results between force plait gait analysis and clinical metrology instruments correlate [14, 20, 33–36], they are less sensitive in evaluating mild lameness [14, 37]. This limitation should be addressed in future studies by comparing clinical metrology instruments results with the examination of these dogs and evaluating lameness. In addition, although handlers were requested to answer the questions honestly, some bias may occur, particularly in cases that could lead to a worse opinion on the handler's work. This could have affected the results. Also, 32.1% of dogs have a previous need of veterinary assistance due to an orthopedic condition. The handlers of these dogs may be more sensitive to detect changes in their dog due to previous experience. They are also more likely to adhere to these types of surveys, as the benefit of proper veterinary assistance has been felt before.

## Conclusions

Our findings show that this population of police working dogs has a good to excellent level of physical fitness. There was a relationship between increasing age, history of orthopedic disease, and worse scores with all questionnaires. All considered questionnaires could differentiate between animals with a previous history of orthopedic disease and sound dogs.

## Methods

In this observational, prospective study, active police working dogs of the Guarda Nacional Republicana were screened (Republican National Guard Canine Unit, Portugal). Inclusion criteria included a bodyweight ≥ 15 kg and an age 1.5–9 years. All animals were active police working dogs, kept in kennels of the Guarda Nacional Republicana, similar in size and construction, in locations spread through Portugal's territory. All dogs were fed the same commercially available dog food (HappyOne High Energy, petMaxi, Portugal), with 28% protein, 15% fat, 8% minerals, and 2.75% fiber.

For each dog, the name, name of the handler, age, sex, breed, specific work, and history of a previous diagnosis of orthopedic disease. The canine handlers completed a digital copy of the COI (available at https://www.vet.upenn.edu/docs/default-source/VCIC/canine-orthopedic-index-weekly-reformat.pdf?sfvrsn=6), HVAS (available online at https://citeseerx.ist.psu.edu/viewdoc/download?doi=10.1.1.174.961&rep=rep1&type=pdf), and FA (available at https://trace.tennessee.edu/cgi/viewcontent.cgi?article=4154&context=utk_gradthes), with as much time as needed to answer all items. The COI contains four questions that sum up to deliver a stiffness score, five questions that provide a gait score, four questions that add up to a function score, and three questions that compose a quality of life score [13]. The overall FA score of an individual dog is calculated by the sum of scores from all questions [17]. A lower score corresponds to better results in both questionnaires, with 0 representing a level of no impairment, scores of 5–7 are classified as showing evidence of borderline impairment, and scores > 8 were classified as functionally impaired [17]. With HVAS, a higher score constitutes a better result.

Normality was assessed with a Shapiro–Wilk test. The Kruskal–Wallis test was used to compare COI, HVAS, and FA scores between breeds, work, age, sex, and history of a previous diagnosis of orthopedic disease. Multiple regression was run to predict COI, HVAS, and FA scores from breeds, work, age, sex, and history of orthopedic disease. Correlations between items were determined with Pearson's correlation. All results were analyzed with IBM SPSS Statistics version 20, and a significance level of *p* < 0.05 was set.

## Data Availability

The datasets used and/or analyzed during the current study are not readily available because the data used in this study is a property of the Guarda Nacional Republicana, a governmental police force from Portugal and, by law, confidential. Access to the datasets is available from the corresponding author on reasonable request.

## Abbreviations

COI: Canine Orthopedic Index
FA: Functional assessment
HVAS: Hudson Visual Analogue Scale
LOAD: Liverpool Osteoarthritis in Dogs
OA: Osteoarthritis
QOL: Quality of Life
